# A Multi-Level Classification Approach for Sleep Stage Prediction With Processed Data Derived From Consumer Wearable Activity Trackers

**DOI:** 10.3389/fdgth.2021.665946

**Published:** 2021-05-28

**Authors:** Zilu Liang, Mario Alberto Chapa-Martell

**Affiliations:** ^1^Ubiquitous and Personal Computing Laboratory, Faculty of Engineering, Kyoto University of Advanced Science, Kyoto, Japan; ^2^Institute of Industrial Science, The University of Tokyo, Tokyo, Japan; ^3^Silver Egg Technology, Osaka, Japan

**Keywords:** sleep tracking, machine learning, consumer sleep technology, wearable sleep trackers, Fitbit, ambulatory sleep monitoring, ubiquitous computing

## Abstract

Consumer wearable activity trackers, such as Fitbit are widely used in ubiquitous and longitudinal sleep monitoring in free-living environments. However, these devices are known to be inaccurate for measuring sleep stages. In this study, we develop and validate a novel approach that leverages the processed data readily available from consumer activity trackers (i.e., steps, heart rate, and sleep metrics) to predict sleep stages. The proposed approach adopts a selective correction strategy and consists of two levels of classifiers. The level-I classifier judges whether a Fitbit labeled sleep epoch is misclassified, and the level-II classifier re-classifies misclassified epochs into one of the four sleep stages (i.e., light sleep, deep sleep, REM sleep, and wakefulness). Best epoch-wise performance was achieved when support vector machine and gradient boosting decision tree (XGBoost) with up sampling were used, respectively at the level-I and level-II classification. The model achieved an overall per-epoch accuracy of 0.731 ± 0.119, Cohen's Kappa of 0.433 ± 0.212, and multi-class Matthew's correlation coefficient (MMCC) of 0.451 ± 0.214. Regarding the total duration of individual sleep stage, the mean normalized absolute bias (MAB) of this model was 0.469, which is a 23.9% reduction against the proprietary Fitbit algorithm. The model that combines support vector machine and XGBoost with down sampling achieved sub-optimal per-epoch accuracy of 0.704 ± 0.097, Cohen's Kappa of 0.427 ± 0.178, and MMCC of 0.439 ± 0.180. The sub-optimal model obtained a MAB of 0.179, a significantly reduction of 71.0% compared to the proprietary Fitbit algorithm. We highlight the challenges in machine learning based sleep stage prediction with consumer wearables, and suggest directions for future research.

## 1. Introduction

Measuring sleep over a prolonged period of time is important for understanding the day-to-day variability in sleep dynamics. Traditional sleep monitoring methods, such as polysomnography (PSG) and videosomnography are expensive, burdensome, and not suited for longitudinal sleep tracking. In contrast, ubiquitous sleep tracking technologies enable unobtrusive measurement of sleep over extended periods in free-living environments. A variety of ubiquitous sleep tracking systems have been developed, including bed sensors (1, 2), wireless EEG (3, 4), smartphone apps (5, 6), and wearable activity trackers (7). Activity trackers are among the most popular consumer sleep tracking technologies. The opportunities and challenges pertaining the personal use of these devices have been well-studied (8–12). In the meantime, recent advances in wearable technology has seen a rise in employing consumer activity trackers, such as Fitbit in research studies for measuring sleep outcomes (13–16). Some studies even leverage Fitbit sleep data as the ground truth to validate new sleep tracking devices (17). The popularity of activity trackers in the research community is mostly due to their appealing features: they are more affordable than medical sleep monitors and easy to use for longitudinal sleep data collection without the necessity of frequent technical support; they have well-designed dashboard to visualize sleep data and offer raw data retrieval at reasonably high granularity through Application Program Interface (API). All these features allure researchers to use these consumer devices as an alternative of medical sleep monitors, especially when research budget is limited and when sleep outcomes are not the main concern.

Nonetheless, there is strong evidence that consumer activity trackers have limited accuracy for measuring sleep (18–25). Previous validation studies show that many devices, such as Fitbit Flex and Fitbit HR Charge tend to overestimate sleep time while underestimate wake time compared to PSG (22, 26). The result was consistent for adolescents (21) and adults (19, 22). Recent validation studies of the latest Fitbit models (e.g., Fitbit Charge 2/3) further revealed that while new models could measure total sleep time and sleep efficiency with satisfactory accuracy, their capability in detecting sleep stages—wakefulness, light sleep, deep sleep and REM sleep—remains to be limited (18, 19). The disparity between Fitbit and medical devices may be more pronounced in free-living environments (18, 27) and among people with sleep problems (28). Furthermore, the accuracy of Fitbit demonstrates temporary patterns, with better accuracy for deep sleep in the first half of a night and better accuracy for REM sleep in the second half of a night (29).

The limitation in measurement accuracy of consumer activity trackers demands the development of new sleep staging algorithms. There is a large body of research on sleep staging with PSG signals, but these algorithms are not readily applicable to consumer activity trackers due to the difference in sensing modalities. A PSG test often involves the measurement of multiple modalities of physiological signals including electroencephalogram (EEG), electrooculogram (EOG), electromyogram (EMG), and electrocardiogram (ECG). In contrast, Fitbit devices only rely on two sensing modalities—accelerometer and PPG—and use these limited signals as input for sleep staging. Although these signals demonstrate sleep stage wise characteristics (30), they are insufficient for achieving satisfactory accuracy in classifying sleep stages (28).

In this study we propose a two-level classification approach for sleep staging with consumer activity trackers. The goal was to train a computational model that captures a mapping relation between the data derived from Fitbit and standard questionnaire to medical-grade sleep staging. To be more specific, the level-I classifier of the model judges whether a Fitbit labeled sleep stage epoch is misclassified, and the level-II classifier reclassifies misclassified epochs. The rationale behind the two-level approach is that a portion of Fitbit labeled sleep stage epochs is correct, and only incorrect labels need to be re-classified. In this study we select Fitbit as a representative of consumer activity trackers because of its popularity, and the two-level classification approach can be generalized to devices of other manufacturers (e.g., Apple Watch, Garmin) with adaptation.

## 2. Methods

### 2.1. Problem Formulation

The objective of this study is to build a computational model for sleep staging based on processed data derived from Fitbit devices. We apply machine learning algorithms because they can discover hidden patterns in complex heterogeneous and high dimensional data (31). The nature of the problem is formulated as follows. Given *x* the feature space containing *M* features, the *x* values are vectors in the form of $x^{v_{1}}$, $x^{v_{2}}$,…,$x^{v_{M}}$, where $x^{v_{m}}$ is either nominal, discrete or continuous feature constructed from the input data. The *y* values are class labels drawn from a discrete set of classes {1, 2, 3, 4}, corresponding to deep sleep, light sleep, REM sleep and wakefulness. A two-level classification model constructs a cascading mapping function f: *y* = *f*_*L*_*II*_(*f*_*L*_*I*_(**x**)) based on *N*^*TR*^ labeled training instances $\left({\text{x}}_{1},y_{1}\right),\left({\text{x}}_{2},y_{2}\right),\ldots ,\left({\text{x}}_{N}^{TR},y_{N}^{TR}\right)$, where *y* belongs to {1, 2, 3, 4}, so that the performance for *N*^*TS*^ new unseen instances can be optimized. The level-1 and level-2 classifiers are denoted as *f*_*L*_*I*_ and *f*_*L*_*II*_. Information related to the training sets and test sets are denoted using superscript notions *TR* and *TS*, respectively.

**Figure 1:**
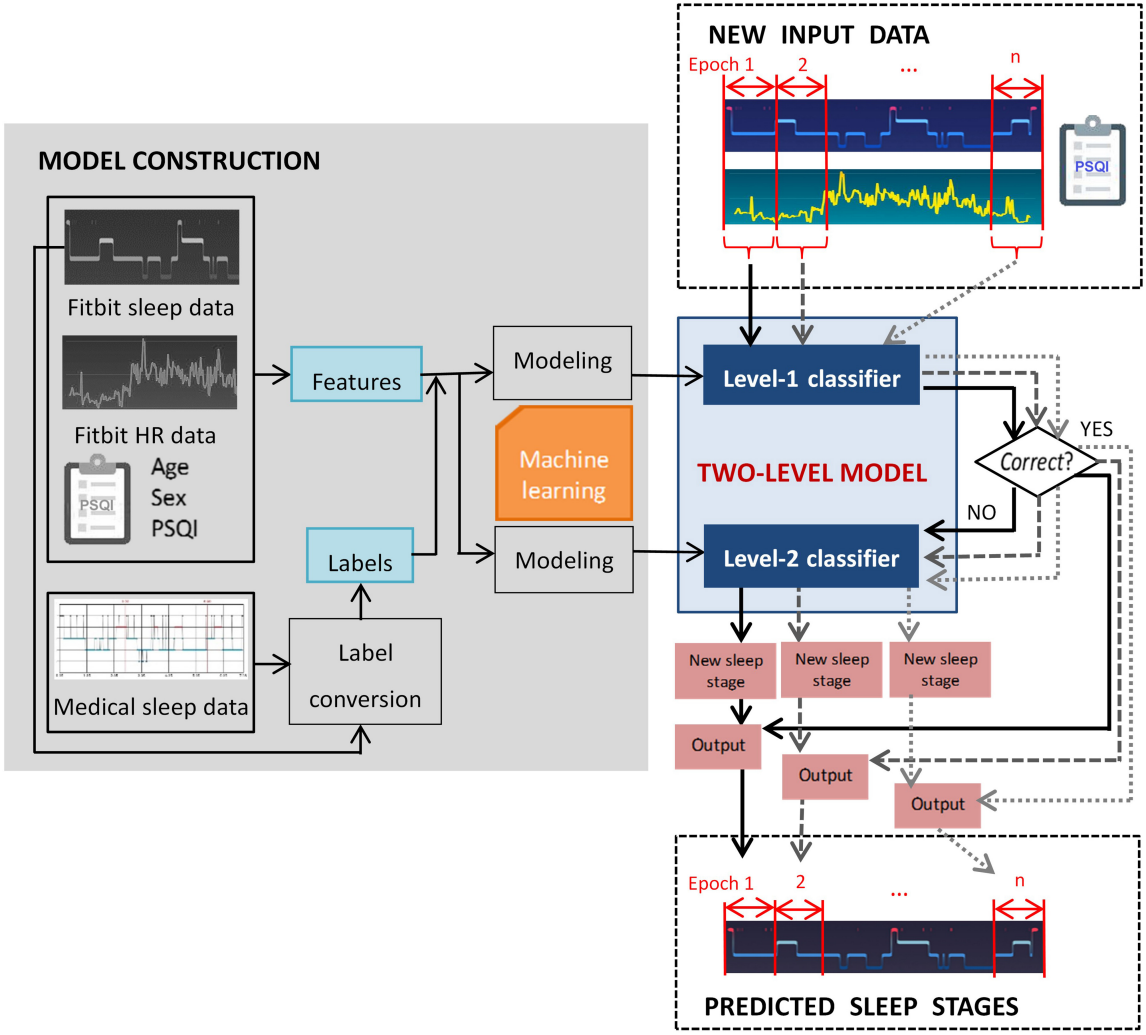
Overview of the proposed two-level classification approach.

In this study, the model performance was evaluated using multiple measures, which will be described in detail in section 2.5. As illustrated in Figure 1, the two-level model performs classification epoch-by-epoch, which is compliant with the standard epoch-wise sleep staging approach in clinical settings. For each epoch, the input data to the two-level model include the corresponding Fitbit labeled sleep stage, aggregated Fitbit sleep metrics for that day, heart rate data, and a few demographic features that can be measured using established psychometric instruments, such as the PSQI questionnaire (32). The output of the model for each epoch is one of the four sleep stages. Epoch-wise predicted sleep labels are aggregated to calculate the total duration of each sleep stage.

### 2.2. Data Collection

We conducted a three-night data collection experiment with 23 healthy adults (nine women; age = 24.8 ± 4.4 years) using a Fitbit Charge 2 and a medical sleep monitor named Sleep Scope. None of the participants had diagnosed sleep problems at the time of the study. Ethics approval was obtained from The University of Tokyo. Data collection was performed in participants' homes to ensure that the distribution of data and noise is representative of real situations in free-living environments. During the data collection experiment, Fitbit Charge 2 was applied to the non-dominant wrist. The electrodes of the Sleep Scope were placed in the middle of the forehead and behind one ear. Details of the two devices are available in Liang and Ploderer (12). Demographic information, such as age, sex, and subjective sleep quality measured by PSQI questionnaire (32) was also collected. The Fitbit data and demographic information are used to construct features while the medical data are used for labeling.

To mitigate the first night effect (33), we used the data of each participant's second night. Nonetheless, if any of the devices had more than 50% of data missing due to technical issues (e.g., electrodes peeling off, device running out of battery), the third night's data were used in place of the second night's. The first night's data were only used when both the second and the third night's data were discarded. Using a web app that we developed in our previous study (15), we retrieved Fitbit labeled sleep stages, aggregated daily sleep metrics, including total sleep time (TST), wake after sleep onset (WASO), sleep efficiency (SE) and the ratio of individual sleep stage, and heart rate data. The Fitbit labeled sleep stage data were retrieved at a granularity of 30 s. The Fitbit heart rate data were retrieved at a granularity of 1 s but in practice the granularity varied. The Sleep Scope generated analysis reports for each selected night, which consists of 30 s-epoch-by-epoch sleep stage data. We wrote a C# program to pre-process the Fitbit labeled sleep stage data, heart rate data and Sleep Scope labeled sleep stage data. All streams of data were firstly synchronized and missing data were handled using interpolation. The Fitbit labeled sleep stages, i.e., “deep sleep,” “light sleep,” “REM sleep,” and “wakefulness,” were respectively mapped to the class “1,” “2,” “3,” and “4.” For Sleep Scope, “stage N3” was mapped to the class “1,” “stage N1,” and “stage N2” were mapped to the class “2,” “stage R” was mapped to the class “3” and “stage W” was mapped to the class “4.”

### 2.3. Feature Construction and Labeling

We constructed 21 features as summarized in Table 1. All features can be derived from the data of Fitbit and PSQI questionnaire without relying on additional information. We selected these features because they have shown to associate to the accuracy of sleep staging (28, 34, 35) and they are easy to derive from the available data. In contrast, other potential features that can be derived from the brainwave signals measured with the medical device and the raw acceleration data measured with Fitbit were not selected as these data are often not readily available in free-living environments.

**Table 1:** Features constructed for epoch *n*.

**Type**	**Feature**	**Collection method**
Static	Age, sex, Pittsburgh sleep quality index (PSQI)	PSQI Questionnaire
	Total sleep time (TST), wake after sleep onset (WASO), sleep efficiency (SE), wake ratio, light sleep ratio, deep sleep ratio, REM sleep ratio	Fitbit
Dynamic	ID of epoch *n*, Fitbit labeled sleep stage for epoch (*n* − 3) − (*n* + 3), average heart rate in epoch *n*, heart rate change in epoch *n* vs. epoch (*n* − 1), heart rate change in epoch *n* + 1 vs. epoch *n*	Fitbit

We divide the features into static features and dynamic features. As shown in Table 1, static features refer to features that demonstrate no intra-individual variability for the selected night. These features include demographic characteristics (i.e., age, sex, and PSQI) and aggregated sleep metrics (i.e., TST, WASO, SE, wake ratio, light sleep ratio, deep sleep ratio, and REM sleep ratio). In contrast, dynamic features are derived from Fitbit time series data and they demonstrate variability from epoch to epoch. These features include epoch ID, Fitbit labeled sleep stage for the current epoch and three preceding and three succeeding epochs, average heart rate in the current epoch, heart rate change in the current epoch against the preceding epoch, and heart rate change in the succeeding epoch against the current epoch.

**Figure 2:**
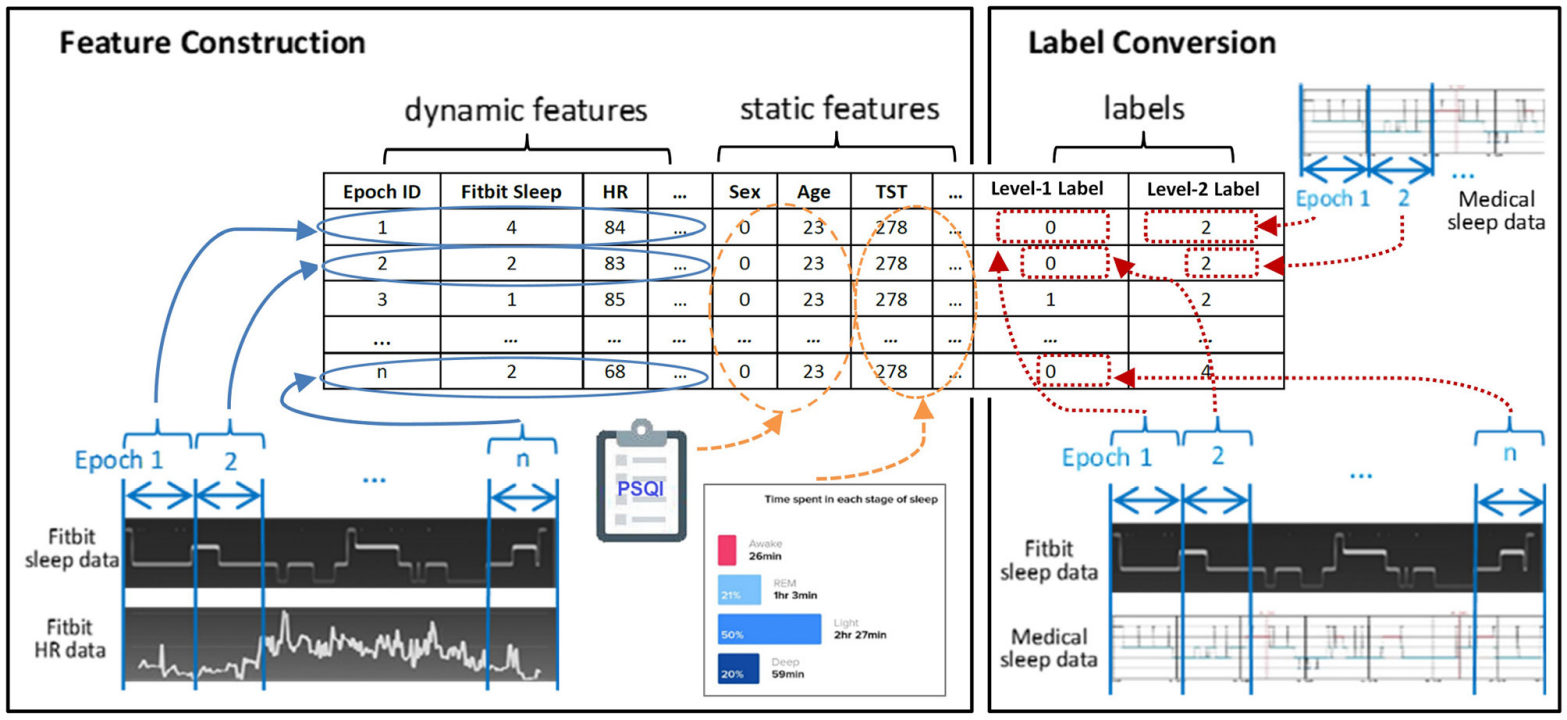
Feature construction and label conversion.

Figure 2 exemplifies the dataset preparation process. We first synchronized the time series data of Fitbit and the medical device to ensure that the data from the two devices matched epoch-wisely. Thereafter we constructed dynamic features from the time series data epoch-by-epoch and each epoch was fed to the dataset as an instance. Lastly, the static features and the labels for two levels were merged with the dynamic features. The same set of features were used at both level-I and level-II classifications. The ground truth labels were obtained epoch-wisely using the following conversion method. For level-I binary classification, the label for each instance was obtained through epoch-wise comparison between the Fitbit and the medical device. If the two devices agreed, the label was 0 (representing correct classification); otherwise, the label was 1 (representing misclassification). For level-II multi-class classification, the medical data was used as the labels. An example of label conversion is shown in Table 2.

**Table 2:** An example of label conversion.

**Epoch ID**	**Fitbit Sleep Data**	**Medical Sleep Data**	**Level-I label**	**Level-II label**
1	4*^a^*	4	0	4
2	4	4	0	4
3	2	4	1	4
4	2	4	1	4
…	…	…	…	…
N-1	3	2	1	2
N	3	3	0	3

a *1 = deep sleep, 2 = light sleep, 3 = REM sleep, 4 = wakefulness*.

### 2.4. Model Construction

We applied different machine learning algorithms at each level of classification. For level-I binary classification, we used three machine learning algorithms—Naïve Bayes (NB), random forest (RF), and support vector machine (SVM) with linear kernel. These algorithms were selected because of their simplicity, computational efficiency, and good performance on binary classification. For level-II classification which involves four classes, we applied the gradient boosting decision tree (XGBoost) because a preliminary analysis of model performance with default parameter values demonstrated their advantage over other widely used machine learning algorithms, such as artificial neural network. Model training of classifier-1 and classifier-2 were independently performed using the same dataset. For each level of classifier, model parameters were tuned through grid search with 10-fold cross validation. Feature normalization was performed in the SVM algorithm.

A major challenge at the level-II classification lies in the imbalanced nature of the datasets, where the class “2” (indicating light sleep) significantly outnumbers other classes. The reason is that a night of normal human sleep typically consists of large portion of light sleep (40–60%), while the portion of REM sleep (15–25%), deep sleep (15–20%), and wakefulness (5–15%) are significantly smaller than light sleep (36). The hitch with the imbalanced nature of sleep data is that standard machine learning algorithms may bias toward the majority class and yield higher learning errors on the minority class. This is especially true for machine learning techniques that are sensitive to class distribution (37). To cope with this problem, we applied resampling techniques to balance different classes. We examined the effect of two simple resampling techniques: random up sampling and random down sampling. Random up sampling randomly generates data points to the minority classes so that the frequency of the minority classes is close to that of the majority one. Random down sampling randomly subsets the majority class to match the frequency of the minority classes. A preliminary study demonstrated the effectiveness of these techniques in enhancing model performance (38). It is worth mentioning that resampling was performed within cross-validation to ensure that only the training sets were resampled, and the test sets were not.

### 2.5. Model Evaluation

We obtained nine models with different combinations of resampling techniques and machine learning algorithms. Models are denoted as *L*1_*L*2_*resamp*_. For example, *NB*_*XGB* denotes the model that uses Naïve Bayes at level-I and XGBoost without resampling at level-II, and *NB*_*XGBd* denotes the model that uses Naïve Bayes at level-I and XGBoost with down sampling at level-II. We compare the performance of the nine two-level models with four baseline models, including the proprietary Fitbit model (denoted as *Fitbit*) and three one-level models (denoted as *XGB*, *XGBu*, and *XGBd*). The three one-level models attempt to reclassify all Fitbit labeled sleep stage epochs through four-class classification instead of selective correction as the two-level models do. These three baseline models all use XGBoost algorithm but with different resampling strategies.

**Figure 3:**
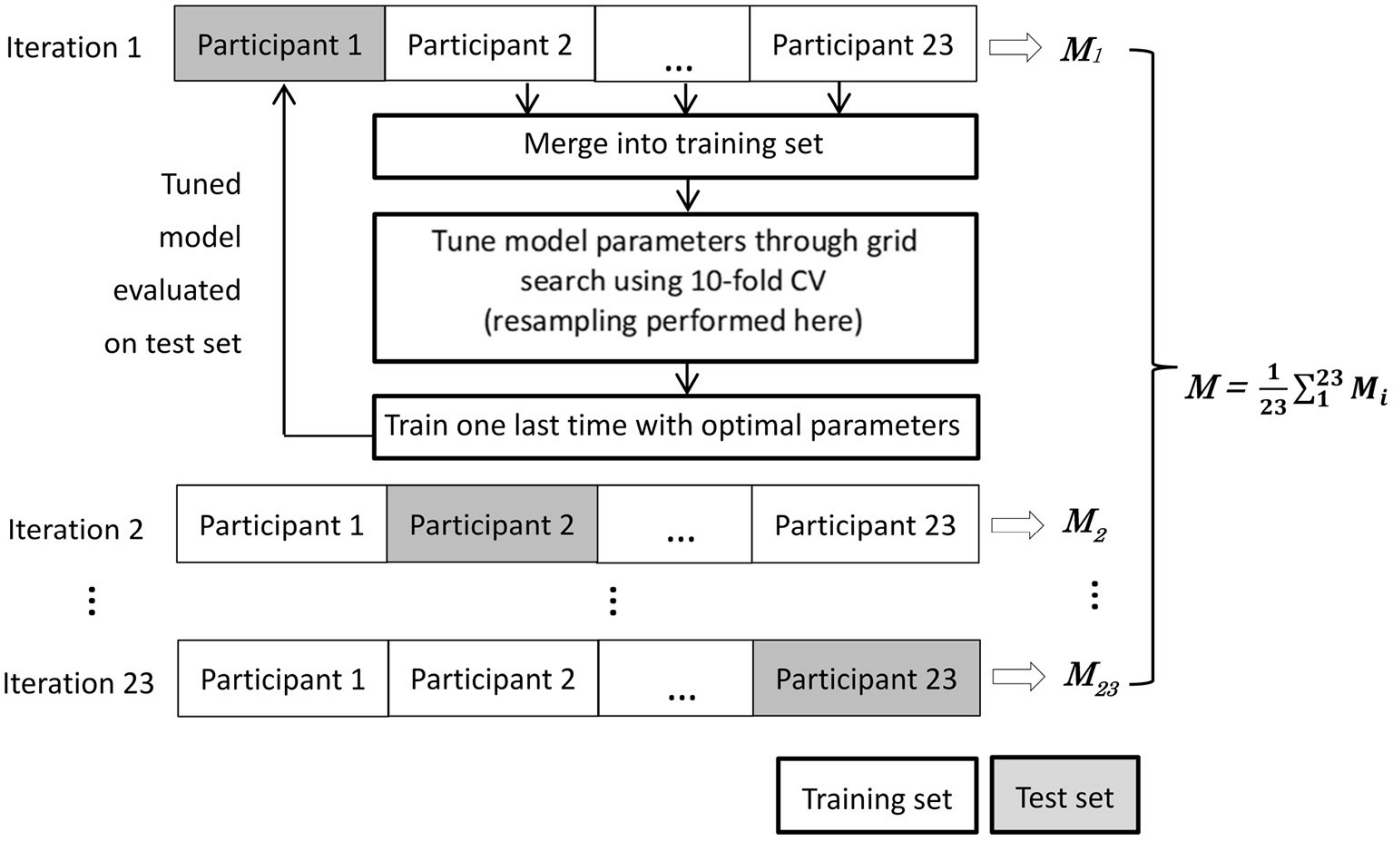
Nested leave-one-subject-out cross validation (LOSO-CV). The ***M*** denotes the set of performance measures.

We evaluate model performance using nested leave-one-subject-out cross validation (LOSO-CV) (39). There are two practical reasons underlying the necessity of using this strategy. First, due to the chronological nature of sleep data, a model should be tested on a whole night of sleep data from one person, not over a mix of epochs from different people. Second, since class distribution demonstrated interpersonal variations, it is important to check the performance of the models on test set with possible data shift, which refers to the cases where training and test data follow different distribution and is especially relevant to imbalanced classification (40, 41). As illustrated in Figure 3, the nested LOSO-CV operates in an iterated manner. In each iteration, the data of one participant are held out as the test set, while the data of the remaining 22 participants are merged and used as the training set. A model is tuned on the training set through intensive grid search using 10-fold cross validation. Once the model is fixed on the training set, it was evaluated on the test set. The nested-evaluation process iterates 23 times with each subject's data being kept out as test set once. We then averaged the performance over all the 23 iterations.

We adopt two types of performance measures–microscopic performance metrics and macroscopic performance metrics–to thoroughly evaluate the two-level models. These measures cover not only epoch-wise comparison between the models and the ground truth (with microscopic measures) but also comparison on aggregated sleep metrics (with macroscopic measures).

#### 2.5.1. Microscopic Measures

Microscopic measures are calculated based on epoch-wise comparison between model predicted value and the true value of each epoch. These measures include overall per-epoch accuracy, Cohen's Kappa, multi-class Mathew's correlation coefficient (MMCC), confusion matrix, correcting power (CP) and over-correcting rate (OR). The per-epoch accuracy and Kappa have been widely used to evaluate the performance of automatic sleep staging algorithms in sleep research. Despite that they may not be the most appropriate performance measure for imbalanced classification, we use them as complementary metric to facilitate cross-study comparison. On the other hand, MMCC is widely accepted as a more appropriate performance measure for imbalanced multi-class classification as it considers the accuracy and error rates on all classes and involves all elements of a confusion matrix (42, 43). The CP and OR are original measures specific to our approach for probing deeper into the behavior of the two-level models. While an ideal model would be characterized by high CP and low OR, in practice it is only feasible to achieve a good trade-off between the two metrics.

- Overall per-epoch accuracy: The number of correctly predicted sleep epochs vs. the total number of epochs. Despite that this metric is not considered as a legitimate performance measure for imbalanced classification in the machine learning community, we use it as a complementary metric that facilitates cross-study comparison.
- Cohen's Kappa: An indicator of model quality by taking into account how much agreement between predicted and true values would be attributed to chance. According to (44), a value <0 indicates no agreement, 0–0.40 as poor, 0.40–0.75 as fair to good, >0.75 as excellent. Higher value indicates better performance.
- Multi-class Matthew's correlation coefficient (MMCC): A single metric that summarizes a confusion matrix and has been considered as one of the best performance measure for multi-class imbalanced classification. A value of −1 and 1 represent perfect misclassification and perfect classification, and a value of 0 indicates random guess (45, 46).
- Confusion matrix: A table that summarizes the prediction accuracy and error rate on individual sleep stages. Each row represents an actual sleep stage, while each column represents a predicted sleep stage.
- Correcting power (CP): The ratio of epochs that are mislabeled by Fitbit and are corrected by the two-level model. Higher value of CP means that more Fitbit misclassified epochs are corrected by the model and hence better model performance.
- Over-correcting rate (OR): The ratio of epochs that are correctly labeled by Fitbit but are misclassified by the two-level model. Lower value of OR indicates better model performance.

(1) $$\begin{array}{l} \Delta =\frac{{\text{M}}_{proposal}-{\text{M}}_{fitbit}}{{\text{M}}_{fitbit}}\times 100\% \end{array}$$

The performance improvement of the two-level models over Fitbit, which is denoted as Δ, is computed using Equation (1), where **M**_*proposal*_ and **M**_*fitbit*_ denote the performance (e.g., accuracy, Kappa, and MMCC) of the two-level models and Fitbit, respectively. Two-tailed *t*-test is applied to examine if statistically significance difference exist between the average performance of the two-level models and that of the Fitbit.

We also perform Pearson's correlation analysis between the performance measures and the class imbalance or the dataset shift to investigate the effect of dataset characteristics on model performance. The Shannon's diversity index (47) is used to quantify the imbalance of individual sleep stage in the test set of each iteration in the LOSO-CV process. A lower value of the Shannon's diversity index implies more imbalance of classes. The Anderson-Darling (AD) statistic (48) is adopted to quantify the difference in sleep stage distribution between a training set and the corresponding test set in each iteration of the LOSO-CV process. A higher value of the AD statistic indicates larger shift between a training set and the corresponding test set.

#### 2.5.2. Macroscopic Measures

Macroscopic measures are calculated by comparing the predicted values and true values on aggregated sleep metrics including the total duration of light sleep, deep sleep, REM sleep, and wakefulness. The following metrics are adopted.

- Bland-Altman (BA) plot (49): A plot that illustrates the difference between two methods as a function of the mean of these methods. The visual examination of the BA plot allows for an evaluation of the global agreement between the model and the ground truth. The biases and lower and upper level of agreement (LOA) are calculated. Two-tailed one sample *t*-test is performed to examine whether the biases are statistically different from 0. A positive bias indicates that the model underestimated a sleep metric against the ground truth, while a negative bias indicates overestimation. The LOAs are computed as bias ±1.96 standard deviation of the differences. Linear fitting with statistical test is performed to examine whether there is a significant tend in errors as a function of the magnitude of the measured value.
- Mean normalized absolute bias (MAB): An average over the normalized absolute values of the bias (i.e., the average discrepancy between a model and the ground truth) of each sleep stage. The MAB of model *j* is calculated using Equation (2), where |*e*_*i,j*_| denotes the absolute value of the bias of model *j* for sleep stage *i*, *min*|*e*_*i*_| and *max*|*e*_*i*_| denote the minimum and maximum absolute bias of all models for sleep stage *i*, and *W, L, D, R* denote wakefulness, light sleep, deep sleep, and REM sleep, respectively.

(2) $$\begin{array}{l} MAB_{j}=\frac{1}{4}\underset{i\in \left\{W,L,D,R\right\}}{\sum }\frac{\left|e_{i,j}|-\min|e_{i}\right|}{\max|e_{i}|-\min|e_{i}|} \end{array}$$

## 3. Results

### 3.1. Descriptive Statistics

The processed dataset consists of 23 nights of sleep data–one night from each participant. Among all the nights of data, five cases used the first night, 13 cases the second night, and five cases the third night. The total sleep time as measured by the medical device ranges from 4.0 to 9.8 h. Complying with the practice in sleep research, each night of sleep was divided into 30 s-epochs, yielding 480–1,176 epochs for individual participant. The demographic information and the sleep characteristics as measured with the medical device and the PSQI are summarized in Table 3. It is worth noting that 12 participants (five females) had a PSQI score higher than or equal to 5, indicating perceived poor sleep quality at the time of the study. These participants referred to their academic stress or the sultry weather as the main reasons of their temporarily poor sleep. In what follows we describe the micro-level and macro-level performance of the models.

**Table 3:** Demographic information and sleep characteristics.

	**Age**	**PSQI**	**Wakefulness**	**Light sleep**	**Deep sleep**	**REM sleep**
			**(min)**	**(min)**	**(min)**	**(min)**
All (*N* = 23)	24.3 ± 2.7	4.3 ± 2.3	55.7 ± 52.9	499.1 ± 149.0	45.1 ± 64.5	177.4 ± 66.1
Female (*N* = 9)	24.4 ± 2.7	4.1 ± 1.5	59.8 ± 72.9	479.8 ± 174.9	58.9 ± 78.5	178.2 ± 66.2
Male (*N* = 14)	24.4 ± 2.8	4.6 ± 2.7	59.1 ± 40.4	537.1 ± 153.1	32.4 ± 50.7	171.8 ± 68.6

### 3.2. Microscopic Performance

**Figure 4:**
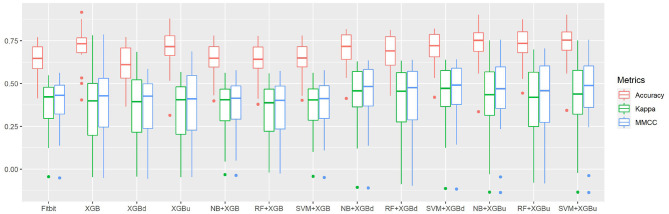
The overall per-epoch accuracy, Kappa and multi-class Matthew's correlation coefficient (MMCC) of sleep staging models.

Figure 4 shows the box plots of the overall per-epoch accuracy, Cohen's Kappa and multi-class Matthew's correlation coefficient (MMCC). The two-level approach demonstrates the best performance when SVM and XGBoost with up sampling are used, respectively for the level-I and level-II classification, yielding an overall per-epoch accuracy of 0.731 ± 0.119 (a 3.4–21.2% increase compared to the four baseline models), Cohen's kappa of 0.433 ± 0.212 (a 14.4–24.1% increase) and MMCC of 0.451 ± 0.214 (a 10.9–16.6% increase). Table 4 presents the performance comparison between Fitbit and the best top six models together with the hypothesis testing results. Two-tailed *t*-test shows that most two-level models achieved statistically significant improvement of per-epoch accuracy. The improvement in Kappa and MMCC was not significant, which is likely due to the relatively small sample size (*N* = 23). A comparison among the three baseline models (i.e., XGB, XGBd, and XGBu) shows that resampling alone does not improve model performance. Adding one level of binary classification to the baseline model with no resampling (i.e., NB + XGB, RF + XGB, and SVM + XGB) reduces the variability but not the average of model performance. In contrast, adding one level of binary classification to the baseline model with resampling successfully improved the model performance. This indicates that resampling at level-II is essential to the performance of the two-level approach.

**Table 4:** Comparison between Fitbit and the best two-level models on microscopic performance measures.

	**Per-epoch accuracy**	**Kappa**	**MMCC**
Fitbit	0.638 ± 0.100	0.371 ± 0.148	0.387 ± 0.151
NBd + XGBd	**0.701** **±** 0.098^*^^,^^a^	0.422 ± 0.175	0.437 ± 0.177
RF + XGBd	0.681 ± 0.109	0.405 ± 0.187	0.419 ± 0.191
SVM + XGBd	**0.704** **±** 0.097^*^	0.427 ± 0.178	0.439 ± 0.180
NBd + XGBu	**0.728** **±** 0.123^**^	0.427 ± 0.214	0.445 ± 0.215
RF + XGBu	**0.722** **±** 0.109^**^	0.414 ± 0.206	0.437 ± 0.200
SVM + XGBu	**0.731** **±** 0.119^**^	0.433 ± 0.212	0.451 ± 0.214

a *Bold indicates statistically significant difference compared to Fitbit*.

* *p < 0.05*;

** *p < 0.01*.

**Figure 5:**
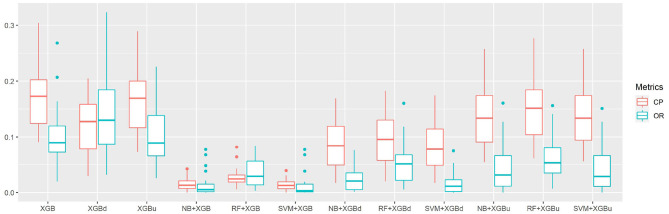
The correction power (CP) and over-correction rate (OR) of sleep staging models.

The confusion matrix for individual sleep stage is provided in Table 5. RF + XGBd achieved the best sleep stage wise performance and improved the average accuracy for both light sleep (by 12.1% compared to Fitbit) and REM sleep (by 14.0%), while minimize the sacrifice on the classification accuracy for deep sleep. In general, down sampling at the level-II classification helped achieved a more balanced performance, whereas up sampling had trivial effect on the overall tendency of the final outcome. With either XGB or XGBu being used for the level-II classification, the models tend to have the best average accuracy for light sleep but deteriorated accuracy for deep sleep and wakefulness. The most common misclassifications were deep/light (two-level model: 42.9–85.3%; Fitbit: 29.1%), REM/light (two-level model: 22.9–41.9%; Fitbit: 31.7%), and wake/light mislabeling (two-level model: 25.7–71.3%; Fitbit: 50.3%). Figure 5 illustrates the correcting power (CP) and over-correcting rate (OR) of the models. The best trade-off between CP and OR is achieved when up sampling was applied at the level-II classification.

**Table 5:** Confusion matrix for sleep stages (%).

		**Ground truth**			
		**Light sleep**	**Deep sleep**	**REM sleep**	**Wakefulness**
Light sleep	Fitbit	**69.3**	29.1	31.7	50.3
	XGB	**87.0**	83.0	41.9	64.4
	XGBd	**61.7**	42.9	22.9	25.7
	XGBu	**85.1**	78.7	41.1	62.0
	NB + XGB	**90.9**	85.3	37.3	70.2
	RF + XGB	**90.2**	82.6	40.1	65.5
	SVM + XGB	**91.2**	85.4	37.1	71.3
	NB + XGBd	**81.0**	52.6	33.8	53.4
	RF + XGBd	**77.7**	49.9	33.1	46.1
	SVM + XGBd	**81.5**	55.5	33.9	54.8
	NB + XGBu	**89.4**	79.4	36.8	68.6
	RF + XGBu	**88.7**	77.9	39.8	63.4
	SVM + XGBu	**89.6**	81.0	36.3	69.3
Deep sleep	Fitbit	21.6	**60.9**	3.5	6.5
	XGB	1.1	**4.4**	0.0	0.4
	XGBd	11.2	**39.6**	1.7	2.3
	XGBu	1.8	**9.3**	0.0	0.4
	NB + XGB	1.0	**3.8**	0.6	0.6
	RF + XGB	1.2	**5.4**	0.2	0.5
	SVM + XGB	0.8	**3.7**	0.0	0.5
	NB + XGBd	9.1	**36.4**	1.5	1.6
	RF + XGBd	9.5	**36.3**	0.9	1.7
	SVM + XGBd	8.9	**34.1**	0.7	1.2
	NB + XGBu	1.8	**9.4**	0.7	0.6
	RF + XGBu	2.0	**10.7**	0.1	0.5
	SVM + XGBu	1.6	**8**.7	0.0	0.4
REM sleep	Fitbit	5.9	2.9	**59.6**	8.1
	XGB	9.5	5.7	**54.5**	12.0
	XGBd	14.2	7.1	**63.9**	18.8
	XGBu	8.6	4.9	**54.6**	12.9
	NB + XGB	6.9	4.0	**59.8**	9.3
	RF + XGB	6.5	5.0	**56.8**	11.3
	SVM + XGB	6.9	4.0	**60.7**	9.3
	NB + XGBd	6.6	3.6	**60.4**	10.6
	RF + XGBd	7.7	5.5	**59.2**	12.3
	SVM + XGBd	6.7	3.3	**61.0**	10.6
	NB + XGBu	7.1	4.2	**60.0**	10.3
	RF + XGBu	6.4	4.5	**56.3**	11.0
	SVM + XGBu	7.1	3.6	**60.9**	10.2
Wakefulness	Fitbit	3.2	2.7	5.3	**35.0**
	XGB	2.4	2.6	3.6	**23.1**
	XGBd	12.9	6.1	11.5	**53.2**
	XGBu	4.5	2.7	4.4	**24.7**
	NB + XGB	1.2	2.4	2.3	**20.0**
	RF + XGB	2.0	2.4	2.9	**22.7**
	SVM + XGB	1.2	2.4	2.2	**19.0**
	NB + XGBd	3.3	3.1	4.3	**34.4**
	RF + XGBd	5.1	4.0	6.8	**39.9**
	SVM + XGBd	2.9	2.8	4.4	**33.4**
	NB + XGBu	1.7	2.4	2.5	**20.5**
	RF + XGBu	2.9	2.5	3.7	**25.0**
	SVM + XGBu	1.6	2.4	2.8	**20.0**

*The bold values indicate the percentage of correctly classified epochs for each sleep stage by different models*.

Table 6 provides the results of the Pearson's correlation analysis between the microscopic performance measures and the Shannon's diversity index of the test sets. Per-epoch accuracy, Kappa and MMCC are in general positively correlated to the Shannon's diversity index. Such correlation is moderate but statistically significant for Fitbit, NB + XGB, RF + XGB, SVM + XGB, RF + XGBd, which implies improved model performance when class distribution in the test sets are more balanced. CP and OR may be weakly or moderately correlated to Shannon's diversity index, but with no statistical significance. Table 7 shows that the AD statistic is negatively correlated to the per-epoch accuracy of almost all models. The correlation strength ranges from moderate to strong and with statistical significance, indicating that prediction errors are more pronounced when there is larger data shift between training set and test set. Kappa and MMCC are not as strongly correlated to the AD statistic as per-epoch accuracy. CP may be moderately and negatively correlated to the AD statistic but the relations are not significant. OR shows strong and positive correlation to the AD statistic for XGBu and SVM + XGBu, indicating increased over-correction rate for these models when there is large drift in data distribution between training set and test set.

**Table 6:** Pearson's correlation coefficients between model performance and Shannon's diversity index (SDI) of test sets.

**Model**	*** $r_{accuracy}^{SDI}$ ***	*** $r_{kappa}^{SDI}$ ***	*** $r_{mmcc}^{SDI}$ ***	*** $r_{cp}^{SDI}$ ***	*** $r_{or}^{SDI}$ ***
Fitbit	**0.46** ^*^ ^a^	**0.48** ^*^	**0.51** ^*^	/	/
XGB	0.35	0.36	0.32	−0.06	−0.06
XGBd	0.36	**0.45** ^*^	0.40	−0.31	−0.23
XGBu	0.38	0.37	**0.41** ^*^	−0.14	−0.25
NB + XGB	**0.46** ^*^	**0.47** ^*^	**0.48** ^*^	0.04	−0.08
RF + XGB	**0.46** ^*^	**0.51** ^*^	**0.51** ^*^	−0.23	−0.30
SVM + XGB	**0.45** ^*^	**0.45** ^*^	**0.46** ^*^	0.12	−0.03
NB + XGBd	0.35	−0.06	−0.04	0.02	−0.00
RF + XGBd	**0.43** ^*^	**0.46** ^*^	**0.46** ^*^	−0.12	−0.24
SVM + XGBd	0.33	0.36	0.37	−0.08	0.33
NB + XGBu	0.39	−0.13	−0.12	−0.04	0.25
RF + XGBu	0.38	**0.46** ^*^	**0.43** ^*^	−0.14	−0.16
SVM + XGBu	0.37	0.40	0.40	0.00	−0.04

a *Bold indicates statistically significant correlation*.

* *p < 0.05*.

**Table 7:** Pearson's correlation coefficients between model performance and Anderson-Darling (AD) statistic.

**Model**	*** $r_{accuracy}^{AD}$ ***	*** $r_{kappa}^{AD}$ ***	*** $r_{mmcc}^{AD}$ ***	*** $r_{cp}^{AD}$ ***	*** $r_{or}^{AD}$ ***
XGB	**−0.65** ^***^ ^a^	−0.36	−0.36	−0.31	0.40
XGBd	−0.40	**−0.45** ^*^	−0.28	−0.07	0.10
XGBu	**−0.72** ^***^	−0.26	**−0.44** ^*^	−0.31	**0.64** ^***^
NB + XGB	**−0.43** ^**^	−0.21	−0.21	−0.05	0.11
RF + XGB	**−0.42** ^*^	−0.23	−0.22	−0.08	0.05
SVM + XGB	**−0.44** ^*^	−0.21	−0.21	−0.00	0.12
NB + XGBd	**−0.65** ^***^	0.10	0.11	0.17	0.04
RF + XGBd	**−0.54** ^**^	−0.29	−0.28	−0.37	0.04
SVM + XGBd	**−0.64** ^**^	−0.31	−0.30	−0.39	0.22
NB + XGBu	**−0.75** ^***^	0.05	0.06	0.03	0.04
RF + XGBu	**−0.67** ^***^	−0.37	−0.34	−0.36	0.36
SVM + XGBu	**−0.76** ^***^	**−0.47** ^*^	**−0.46** ^*^	−0.36	**0.81** ^***^

a *Bold indicates statistically significant correlation*.

* *p < 0.05*;

** *p < 0.01*;

*** *p < 0.001*.

### 3.3. Macroscopic Performance

**Figure 6:**
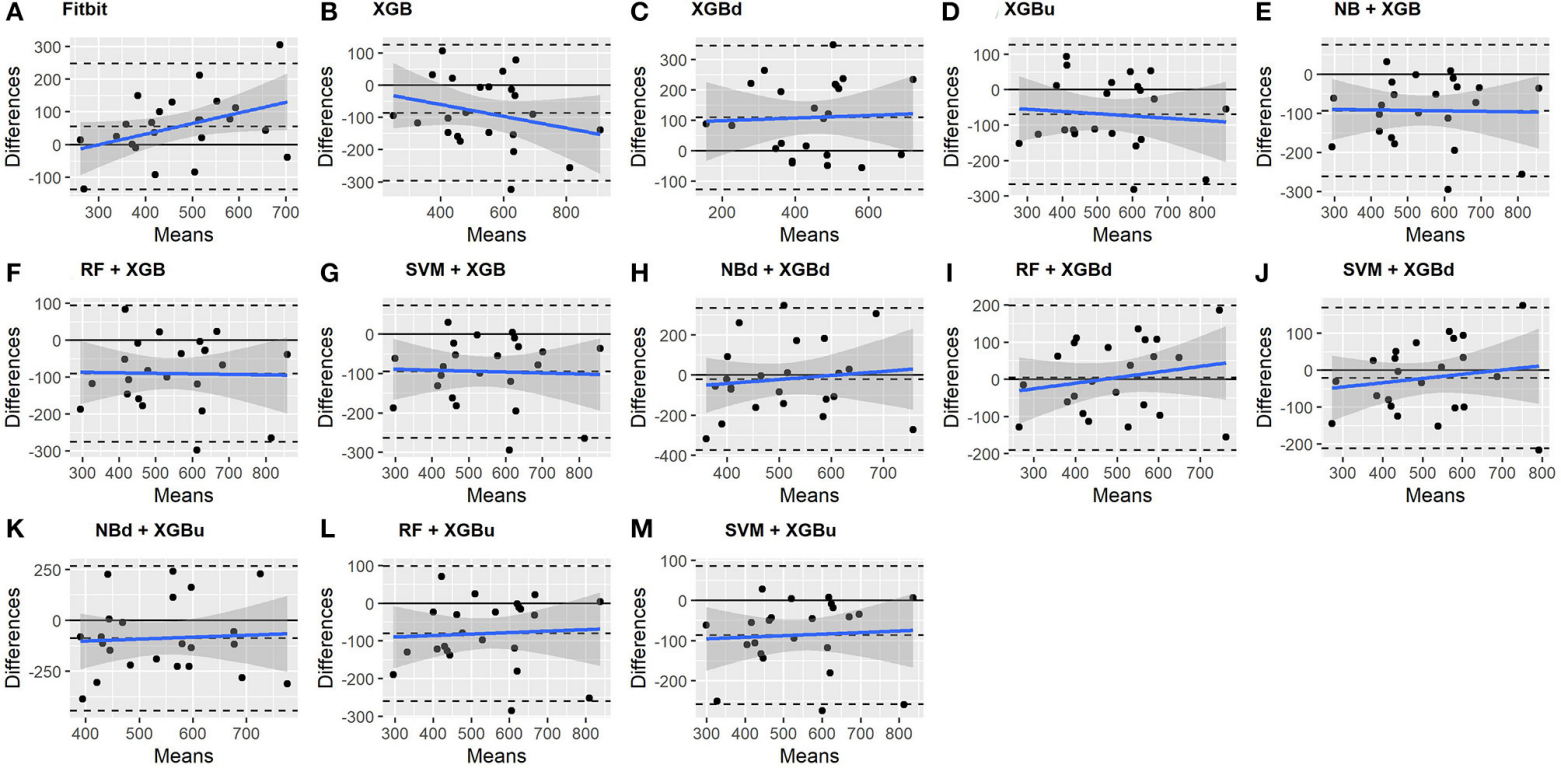
Bland-Altman plots for light sleep.

**Figure 7:**
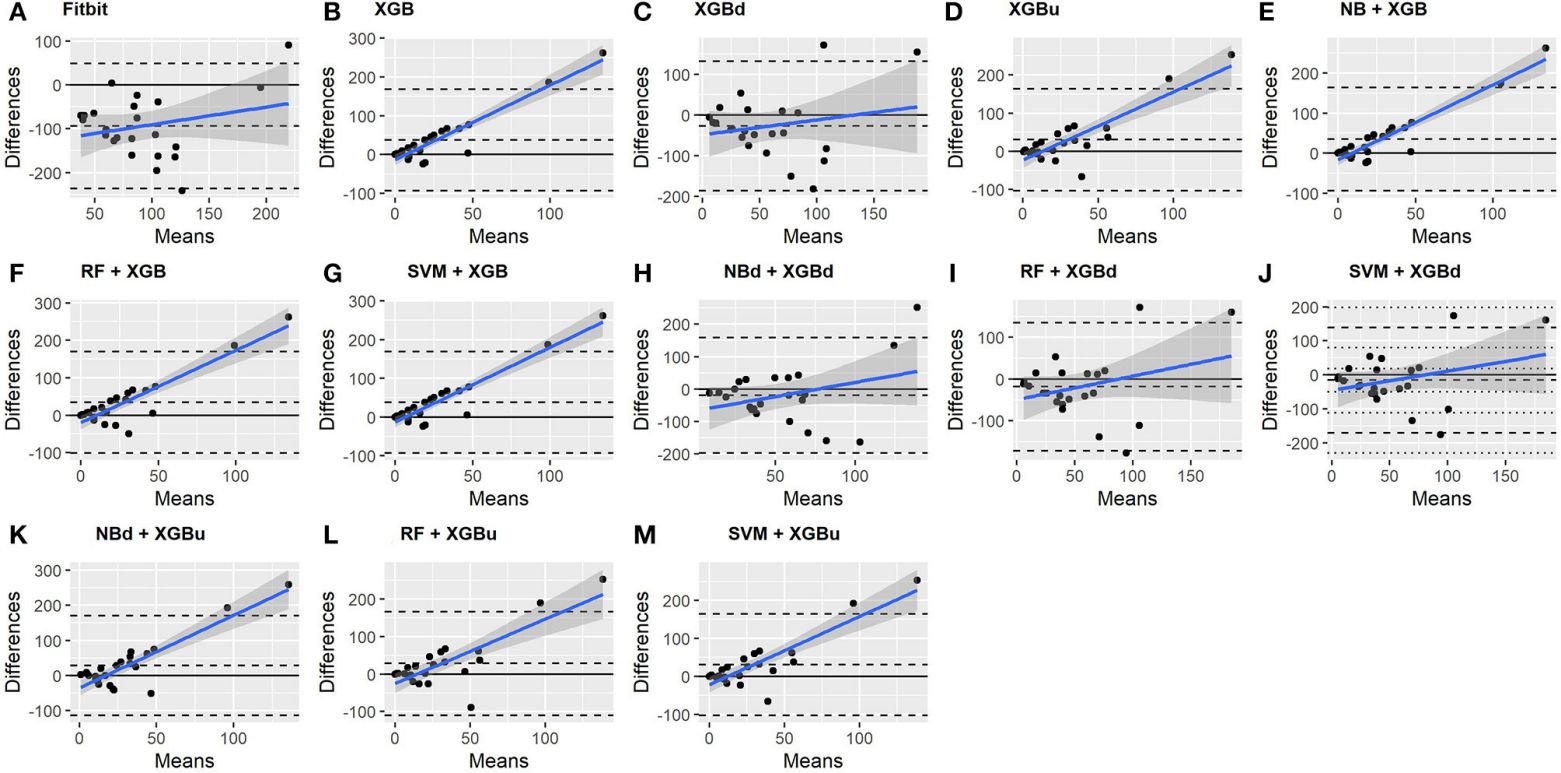
Bland-Altman plots for deep sleep.

**Figure 8:**
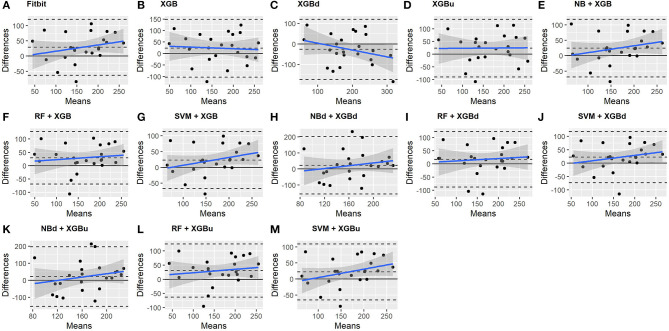
Bland-Altman plots for REM sleep.

**Figure 9:**
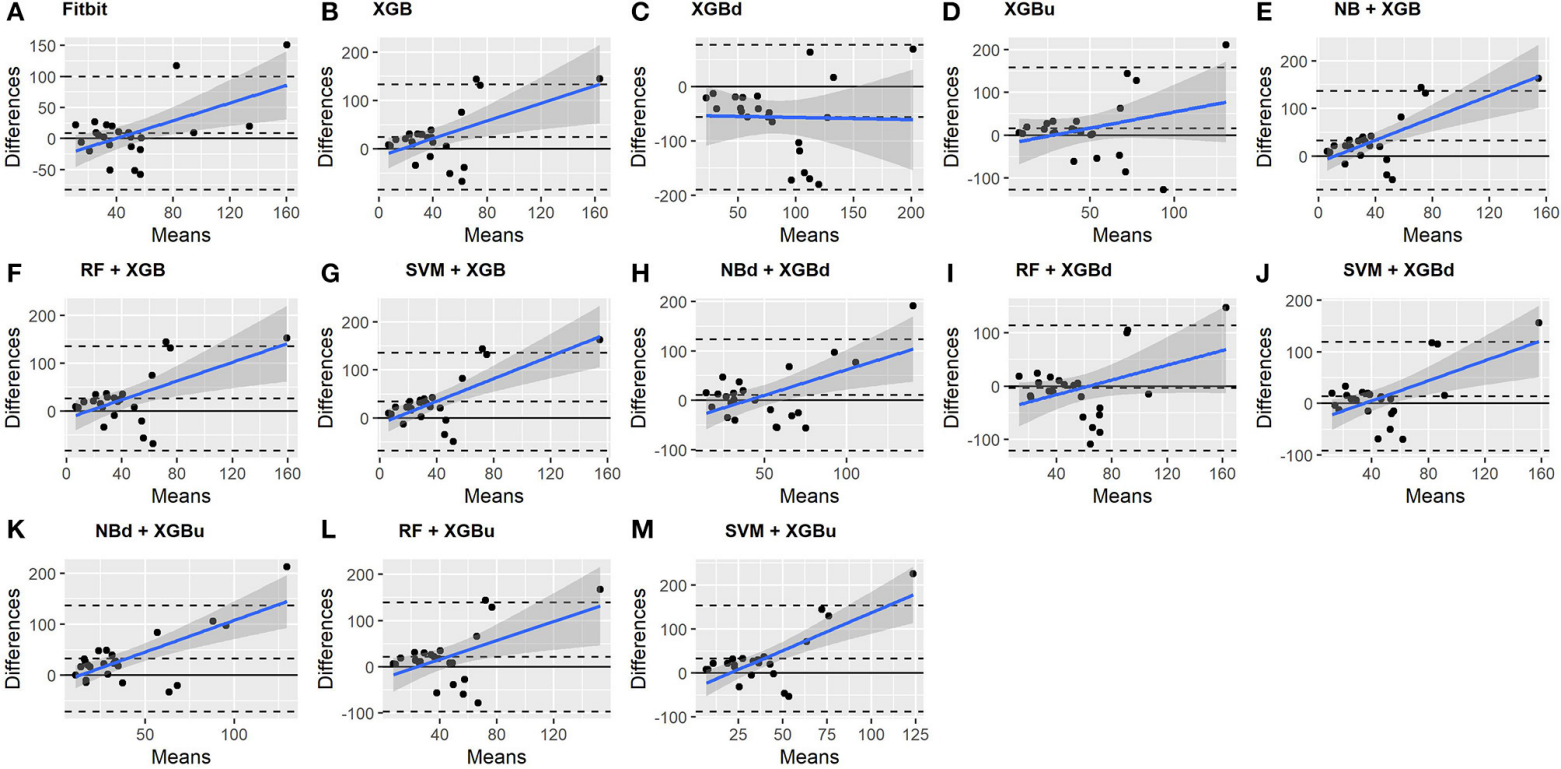
Bland-Altman plots for wakefulness.

The Bland-Altman plots constructed for the individual sleep stage are provided in Figures 6–9. Figure 6 shows that the two-level model RF + XGBd (mean bias: 4.7 min, bias to limit: 194.9 min; *t* = 0.224, *p* = 0.825) agreed well to the ground truth for light sleep. Two other models, NBd + XGBd (mean bias: −19.7 min, bias to limit: 353.5 min; *t* = −0.525, *p* = 0.605) and SVM + XGBd (mean bias: −20.9 min, bias to limit: 190.8 min; *t* = −1.030, *p* = 0.314), also had smaller mean bias compared to Fitbit (mean bias: 55.9 min, bias to limit: 192.0 min; *t* = 2.737, *p* = 0.012). Statistical test on the linear fitting between model difference and model mean showed no significant trend in all models.

All two-level models have better agreement to the ground truth for deep sleep compared to the baseline models. The best agreement was achieved by SVM + XGBd (mean bias: −15.3 min, bias to limit: 154 min; *t* = −0.929, *p* = 0.363), followed by RF + XGBd (mean bias: −18.5 min, bias to limit: 153.8; *t* = −1.129, *p* = 0.271) and NBd + XGBd (mean bias: −18.8 min; bias to limit: 178.0 min; *t* = −0.991, *p* = 0.332). These three models together with Fitbit demonstrated no statistically significant trend. In contrast, a positive trend was observed in other models. Better agreement was observed for nights with shorter deep sleep, and on nights when there was longer deep sleep the models were more variable. We also noticed that the scatter around the bias line gets larger as the average gets higher.

As for REM sleep, RF + XGBd (mean bias: 17.5 min, bias to limit: 105.12 min; *t* = 1.567, *p* = 0.238) achieved the smallest bias to the ground truth among all models. SVM + XGBu (mean bias: 22.0 min, bias to limit: 87.3 min; *t* = 2.372, *p* = 0.027) and SVM + XGBd (mean bias: 22.3 min, bias to limit: 95.4 min; *t* = 2.197, *p* = 0.039) also had better performance than Fitbit (mean bias: 29.1 min, bias to limit: 92.0 min; *t* = 2.971, *p* = 0.007). No model showed statistically significant trend in model difference as a function of model mean.

As for wakefulness, RF + XGBd (mean bias: −3.7 min, bias to limit: 117.3 min; *t* = −0.296, *p* = 0.770) is the only model that achieved better agreement to the ground truth compared to Fitbit (mean bias: 8.7 min, bias to limit: 90.9; *t* = 0.894, *p* = 0.381). Statistically significant and positive trend is evident along the graph for all models except XGBd, XGBu, and RF + XGBd, and the scatter around the bias line gets larger as the average gets higher for all models.

**Figure 10:**
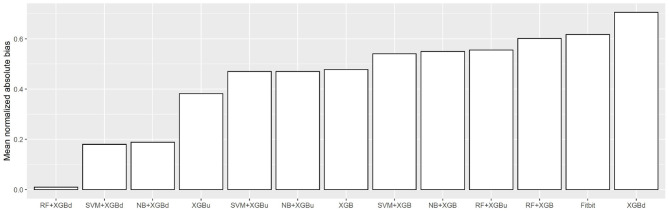
Mean normalized absolute bias (MAB).

Figure 10 shows the mean normalized absolute bias (MAB) of all models. RF + XGBd achieved the lowest MAB (0.010), which translates to a 98.4% decrease compared to that of Fitbit (0.617). All two-stage models achieved lower MAB than Fitbit. Breaking down into the four sleep stages, it shows that the decrease in MAB in the two-level models relied on all sleep stages, especially the deep sleep stage.

## 4. Discussion

### 4.1. Principal Findings

This study demonstrates the feasibility of achieving reasonable accuracy in sleep staging with processed data derived from consumer wearables. We have described the evaluation results of the two-level models using multiple performance measures. The two-level models significantly outperform the baseline models in multiple facets when validated against a medical-grade single channel portable EEG. The best microscopic and macroscopic performance were achieved, respectively by SVM + XGBu and RF + XGBd, and SVM + XGBd achieved a good trade off between microscopic and macroscopic performance. Breaking down into individual sleep stage, the best accuracy of the three models was obtained for light sleep (i.e., 89.6%), whereas prediction accuracy for deep sleep was deteriorated. The two-level models also demonstrated different tendency in misclassification compared to Fitbit. Less light sleep, REM sleep and wake epochs were misclassified as deep sleep, and less REM sleep epochs were misclassified as wakefulness. Nevertheless, more deep sleep and REM sleep epochs were misclassified as light sleep, and more wakefulness epochs were misclassified as REM and light sleep. This indicates that the features constructed using the processed data of the consumer activity tracker may not be sufficient to capture the differences between certain sleep stage pairs.

Regarding overall agreement, the two-level models achieved satisfactory agreement with the ground truth for light sleep and wakefulness. They had lower discrepancy for deep sleep and REM sleep compared to Fitbit, while also eliminating the trends in bias for light sleep and REM sleep. Nevertheless, inconsistent variability was still observed for deep sleep and wakefulness. The scatter around the bias line gets larger as the average gets higher, but such tendency is weaker than Fitbit. Taking all four sleep stages together, the two-level models achieved a MAB of as low as 0.010, which is <1% of the MAB of the baseline models.

We also observed the effect of imbalanced data and dataset shift on model performance. Higher degree of class imbalance of the test set (as quantified by lower value of the Shannon's diversity index) was significantly correlated to deteriorated model performance in multiple dimensions. Resampling at the level-II classification of the two-level models significantly enhanced the overall model performance. While the two-level models using no resampling at level-II performed similarly to or even worse than one level models, both down sampling and up sampling at level-II help improve the model performance with significantly enhanced correcting power. Up sampling generally enhanced microscopic performance, and down sampling allows the models to achieve more balanced accuracy for individual sleep stages and better macroscopic performance. On the other hand, larger dataset shift (as quantified by higher value of the Anderson-darling statistic) was also significantly correlated to deteriorated model performance. The effect was especially manifested in models with up sampling. This is likely due to the side effect of up sampling, which affected the distribution of the three minority classes (while down sampling only affecting one major class) and thus exacerbated dataset shift.

### 4.2. Comparison to Prior Work

While automatic sleep staging with PSG signals has been well-studied for more than two decades, research efforts on approximating sleep stages with widely available consumer activity devices has just started a few years ago.

To our knowledge, there are only three publications attempting four-class sleep stage classification with embedded sensors in consumer activity trackers (35, 50, 51). Fonseca et al. developed a model using features extracted from ECG heart rate variability and trained with multi-class Bayesian linear discriminant (142 features). This model achieved a Kappa of 0.42 ± 0.12 and overall per-epoch accuracy of 59% (51). This model overestimated wakefulness (mean bias = 3.2 min), light sleep (mean bias = 28.3 min), underestimated REM sleep (mean bias = 41.5 min), and agreed well for deep sleep, which yielded a mean absolute bias of 18.3 min. Beattie et al. developed models based on raw acceleration and PPG signals from activity trackers using linear discriminant classifiers, quadratic discriminant classifiers, random forest and support vector machine. These models achieved a Cohen's Kappa of up to 0.52 ± 0.14 and overall per-epoch accuracy of up to 69% (50). The model agreed well to the ground truth for deep sleep and wakefulness, whereas underestimated light sleep (mean bias = 11.1 min) and overestimated REM sleep (mean bias = 13.0 min). The most common misclassifications were light/REM and light/wake mislabeling. This echoes the finding in the present study that the pairs of light/REM and light/wakefulness remains to be challenging to classify based on data from consumer wearable activity trackers. In a recent study, Walch et al. developed sleep staging models using several machine learning algorithms with raw signals from smart watches (35). With artificial neural network, the model achieved the best performance of overall per-epoch accuracy = 72% and Kappa = 0.30.

In contrast with the above models that perform sleep classification with raw sensory signals (e.g., acceleration and PPG signals), our approach relies only on processed data from wearable activity trackers (e.g., steps, heart rate, sleep metrics). This strategy sacrifices the granularity of the input data and has to cope with the noise introduced by the proprietary algorithms that are used to process the raw signals, but may increase the feasibility for the models to be implemented in practice, as many wearable devices do not provide public API for retrieving raw sensory signals (15). Our two-level approach adopts a “selective correction” strategy that only reclassifies mislabeled sleep epochs by the manufacturer's proprietary model while keeps labels of the epochs that are correctly classified. The evaluation results demonstrate comparable performance of the two-level models against existing models. That said, there is still much room for improvements especially regarding the accuracy for deep sleep and wakefulness. Possible directions include expanding the feature extraction window, using different set of features at each level of classification, and addressing the class imbalance and dataset shift problems with more advanced techniques.

### 4.3. Challenges in Machine Learning Based Sleep Stage Prediction With Consumer Wearable Activity Trackers

Despite of using only a few manually crafted features, the two-level models demonstrate promising performance. Enhancing the accuracy of widely used consumer sleep technologies not only brings benefits to the end users by helping them gain a better understanding of their sleep health, but also enables researchers to use these devices to measure sleep outcomes conveniently in longitudinal studies.

As consumer activity trackers take over medical actigraphy with more sensing modalities and functions (52), the opportunities for these devices to be used as a complementary tool in sleep medicine have been increasing (53, 54). Nonetheless, there is a need to develop new algorithms, and machine learning is a powerful building block. In the meantime, the present work as well as previous studies highlight several challenges in applying machine learning to sleep stage prediction with consumer devices. Addressing these issues requires inputs from multiple disciplines including sleep medicine, data science, statistics and the consumer wearable industry.

#### 4.3.1. Challenge 1: Class Imbalance Due to Intrinsic Sleep Stage Distribution in Human Sleep Structure

Previous sleep studies have shown that the portion of individual sleep stage in a normal night of sleep is highly imbalanced, with light sleep dominating more than 50% of the total sleep duration (36), which is supported by the sleep data collected in the present study. Applying machine learning algorithms to sleep staging thus face the challenge of class imbalance. Standard machine learning algorithms may bias toward light sleep and yield high learning errors on other sleep stages. Many solutions have been developed to handle the imbalanced problem in the machine learning community. In this study we investigated the effectiveness of two basic resampling techniques. Down sampling at the level-II classification helped achieve more balanced performance for individual sleep stage and significantly reduced systematic bias. Nevertheless, the accuracy for minority classes (especially deep sleep) still has large room for improvement. A possible reason could be that the basic resampling techniques introduced extra noise to the data by affecting the variance of the features, which exacerbated dataset shift and adversely affected classification accuracy. Future studies may explore advanced techniques for handling imbalanced dataset, such as ensemble methods (54) and cost-sensitive learning (55).

#### 4.3.2. Challenge 2: Dataset Shift Due to Intra/Inter-Individual Variability in Sleep Structure

Another issue closely related to dataset characteristics is the dataset shift problem which refers to the possible differences in the data distribution for training and test data (40, 56). Data shift is a pertinent issue in sleep staging due to day-to-day and inter-individual variability in sleep structure (57, 58). This has been confirmed in the present study using the AD statistic. Dataset shift invalidates the assumption of many supervised learning algorithms that the joint probability distribution remains unchanged between training and testing. It could be a major reason for the high variability of the model performance across participants in this study and in Walch et al. (35). While the issue of dataset shift was not exclusively addressed in this study, future research may employ methods, such as importance-weighted cross validation (IWCV) (59), subclass re-estimation (60), GP-based feature extraction (61) and transfer learning (62–64).

#### 4.3.3. Challenge 3: Lacking a Framework for Model Performance Evaluation

Machine learning based sleep staging also face the challenge of lacking a framework for model performance evaluation. No consensus has been reached so far as to which measures yield the most objective and unbiased evaluation on sleep staging models. This study evaluates model performance using three measures, which demonstrates slightly different results with varied level of statistical significance. While performance measures, such as overall per-epoch accuracy and Cohen's Kappa have been routinely used in previous studies, these measures are not considered as legitimate for imbalanced classification in the machine learning community. Performance measures, such as MMCC adopted in this study may be a promising alternative, but a cut-off value for good performance is yet to be decided. Furthermore, the performance evaluation in this study assumed equal importance of all sleep stages, whereas in practice a certain sleep stage may hold more clinical significance than other sleep stages. For example, the diagnosis of narcolepsy requires the accurate detection of REM sleep. To this end, a universal framework for model performance evaluation should be able to accommodate a wide range of application scenarios.

### 4.4. Limitations of Present Study

The present study has two major limitations. First, a single channel medical EEG device was used as the reference instead of the gold standard PSG. Data recorded using this device may contain noise that negatively affected the supervised model training process. Second, the robustness of the model performance on minor classes–especially deep sleep and wakefulness–demands further improvement. While the macroscopic performance of the two-level models demonstrated good agreement for light sleep, REM sleep and wakefulness, epoch-wise inspection showed that improvement in microscopic performance was limited to light sleep. It was also observed that the model performance heavily relied on the choice of the machine learning techniques and the parameter tuning. These are likely the consequence of the data imbalance and dataset shift issues, which was further exacerbated by the relatively small size of the training dataset. Our further research will focus on addressing these limitations through improving research design (e.g., increasing sample size and using PSG data as the ground truth) and applying advanced machine learning techniques (e.g., online machine learning and transfer learning).

## Data Availability Statement

The datasets presented in this study can be found in online repositories. The names of the repository/repositories and accession number(s) can be found at: https://github.com/PiranitaGomez/Fitbit_sleepStaging_multiLevelML.

## Ethics Statement

The studies involving human participants were reviewed and approved by School of Engineering, The University of Tokyo. The patients/participants provided their written informed consent to participate in this study.

## Author Contributions

ZL conceived and designed the study, performed the data collection experiments, model training and testing, and drafted the paper. MC-M retrieved the Fitbit data, performed the data pre-processing, and made the revision. All authors contributed to the article and approved the submitted version.

## Conflict of Interest

The authors declare that the research was conducted in the absence of any commercial or financial relationships that could be construed as a potential conflict of interest.
